# Ellipsoidal mirror for two-dimensional 100-nm focusing in hard X-ray region

**DOI:** 10.1038/s41598-017-16468-1

**Published:** 2017-11-27

**Authors:** Hirokatsu Yumoto, Takahisa Koyama, Satoshi Matsuyama, Yoshiki Kohmura, Kazuto Yamauchi, Tetsuya Ishikawa, Haruhiko Ohashi

**Affiliations:** 1 Japan Synchrotron Radiation Research Institute, 1-1-1 Kouto, Sayo-cho, Sayo-gun, Hyogo, 679-5198 Japan; 2 Department of Precision Science and Technology, Graduate School of Engineering, Osaka University, 2-1 Yamada-oka, Suita, Osaka, 565-0871 Japan; 3 RIKEN SPring-8 Center, 1-1-1 Kouto, Sayo-cho, Sayo-gun, Hyogo, 679-5148 Japan

## Abstract

Cutting-edge hard X-ray microscopy strongly depends on sophisticated focusing optics and ultrabright X-ray sources at synchrotron-radiation and X-ray free-electron laser (XFEL) facilities. These facilities typically provide two-dimensional nanofocusing X-ray beams by combining one-dimensional focusing mirrors. However, single-reflecting two-dimensional focusing mirrors with an ellipsoidal surface, which are well-known to possess high efficiency, have limited microfocusing applications. In this paper, we present an ultrahigh-precision ellipsoidal mirror for two-dimensional X-ray nanofocusing by overcoming the difficulties faced in the manufacturing process of its aspherical surface, including the surface-processing methods and surface metrology. The developed mirror has nanoscale accuracy, and it achieves focus size of 85 nm × 125 nm (full width at half maximum) using 7-keV X-rays. Two-dimensional focus was demonstrated in the same focal plane by resolving 50-nm test structures by scanning X-ray microscopy using a focusing beam. These achievements represent an important first step toward realizing two-dimensional aspherical mirrors with complex designs, in addition to ultralow loss and unprecedented small focusing property for extensive optical applications in synchrotron-radiation and XFEL facilities as well as in other scientific fields that require ultraprecision optical surfaces.

## Introduction

Owing to constant developments in high-resolution microscopy, X-ray analytical techniques have become essential tools in various fields of science in which focusing optics play an important role^1,2^. Among various types of focusing optics, total-reflection mirrors afford tremendous advantages in terms of achromaticity and high efficiency relative to other optics that use diffraction or refraction phenomena. Following the first observation of the total reflection of X-rays from a plane mirror surface by Compton in 1923^3^, total-reflection mirrors were used by Kirkpatrick and Baez in 1948^4^ to obtain the first two-dimensional X-ray image. The so-called Kirkpatrick‒Baez (K‒B) geometry with two concave mirrors in an orthogonally crossed geometry is still typically used for moderate or micro-/nano-two-dimensional focusing optics^5,6^ to illuminate samples in synchrotron-radiation and X-ray free-electron laser (XFEL) facilities^7,8^. At present, the minimum focusing beam size for total-reflection mirror optics is 25 nm [full width at half maximum (FWHM)] using a one-dimensional focusing mirror^9^. Nanoscale two-dimensional focus with nearly diffraction-limited performance can be realized by simply combining two one-dimensional focusing mirrors with an elliptical-cylinder shape^10–13^.

The manufacturing process of mirror optics, such as surface-machining methods and surface metrology, has improved steadily^11,12,14–26^. However, ideal two-dimensional nanofocusing mirrors with an ellipsoid revolution in the soft and hard X-ray regions remain exceedingly difficult to manufacture because of their complex and aspherical shape with nanoscale accuracy and atomically controlled surface roughness^27–29^. Studies are aiming to manufacture mirrors with an ellipsoid revolution that can realize a focal spot size of the order of a few nanometers without chromatic aberration^30^.

**Figure 1:**
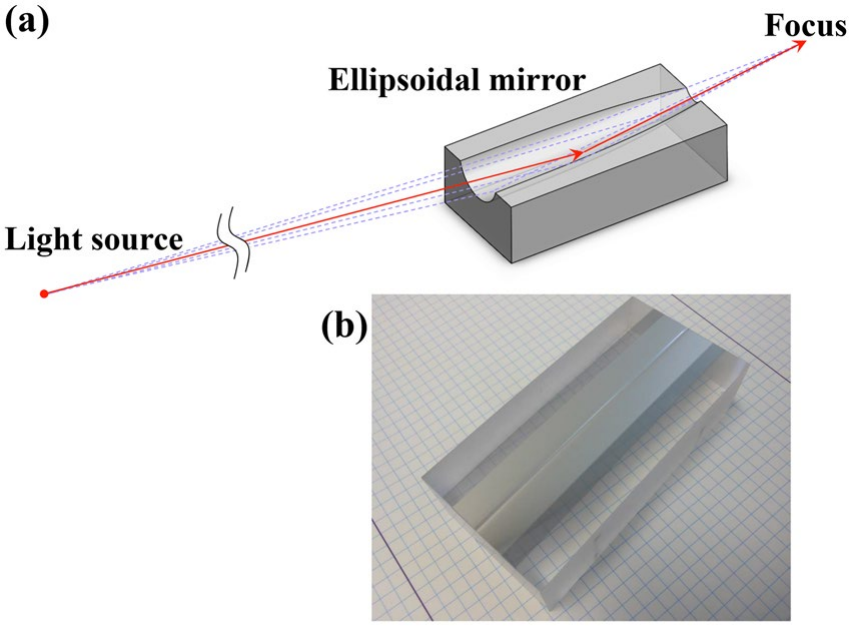
Ellipsoidal focusing mirror under grazing-incidence angle conditions. (**a**) Schematic view. (**b**) Photograph of the developed ellipsoidal focusing mirror. The mirror substrate has dimensions of 100 mm × 50 mm × 30 mm. An ellipsoidal shape is formed on a glass substrate surface. The mirror surface was coated with a platinum thin film.

Our group aimed to manufacture simple focusing mirrors with an ellipsoidal shape (see Fig. 1) that do not have a 360° rotational form but can two-dimensionally focus X-rays with a single reflection, to depart from the traditional K‒B geometry. Ellipsoidal focusing mirrors allow a small diffraction-limited focus size and high-density focus with the design of a large spatial aperture in the sagittal focusing direction, compared with that in the meridional focusing direction. These characteristics are particularly advantageous and are not available in mirrors with K‒B geometry. Nevertheless, even the manufacture of nanofocusing ellipsoidal mirrors is extraordinarily challenging. Currently, toroidal mirrors are used for simplified two-dimensional focusing optics for a micrometer beam size in the soft and hard X-ray regions.

Our group has been developing direct fabrication techniques for one-dimensional nanofocusing mirrors with an elliptical-cylinder shape^22–26^. To address the problem of fabricating two-dimensional aspheric mirrors, we developed surface-processing techniques and a surface-metrology system for nanofocusing ellipsoidal mirrors. A pilot study was conducted to examine the accuracy of the metrology with a partly manufactured mirror using hard X-rays^31,32^.

The present study aims to realize a two-dimensional 50-nm-resolution ellipsoidal focusing mirror in the hard X-ray region. The focusing performance of the manufactured ellipsoidal mirror was evaluated in the synchrotron-radiation facility of SPring-8, Japan. The focusing beam size was measured as 85 nm × 125 nm (FWHM); this is the best result achieved using ellipsoidal mirrors. Furthermore, 50-nm test patterns were resolved using a focusing beam. Thus, we have realized a significant advancement in mirror optics.

## Results

### Manufacturing results of ellipsoidal focusing mirror

An ellipsoidal mirror for nanofocusing was designed for total reflection in the hard X-ray region in a synchrotron-radiation facility. Figure 1(a) shows a schematic of the ellipsoidal focusing mirror under grazing-incidence angle conditions. Figure 1(b) shows a photograph of the developed ellipsoidal focusing mirror. The glass substrate surface of the mirror is coated with a platinum thin film. The optical design and manufacturing results of the developed mirror are listed in Table 1 and presented in the Methods sections (“Properties of ellipsoidal focusing mirror” subsection). The diffraction-limited focus sizes are estimated to be 37 and 67 nm (FWHM) in the meridional and sagittal focusing directions, respectively, at X-ray energy of 7 keV with an effective mirror area and numerical apertures (N.A.), as listed in Table 1. The manufactured mirror’s spatial acceptance in the sagittal focusing direction is approximately half that in the meridional one. The N.A. in the sagittal focusing direction is not large enough to produce a smaller sagittal focus size with the diffraction-limited performance than in the meridional one. The surface metrology used in the manufacturing limits the sagittal N.A. The mirror has an ideal reflectivity of 75% at X-ray energy of 7 keV. A surface-figure accuracy of 1 nm (RMS) and a 2.5-nm [peak-to-valley (PV)] height must be satisfied in the designed optics for diffraction-limited focusing performance. These values are derived from a wave-optical calculation^33^ and Rayleigh’s quarter wavelength rule^34^, respectively.

**Table 1:** Optical parameters of developed mirror.

Parameter	Conditions
Surface profile	Ellipsoid
Substrate material	Synthetic fused silica
Surface coating	Platinum 50 nm
Mirror substrate size	100 mm × 50 mm × 30 mm
Effective mirror dimension:	
in the meridional focusing direction	93 mm
in the sagittal focusing direction	0.45 mm
Grazing-incidence angle at the mirror center	9.0 mrad
Focal length:	
light source to mirror center	50 m
mirror center to focus	200 mm
Semimajor axis	25.10 m
Semiminor axis	28.46 mm
Spatial acceptance:	
in the meridional focusing direction	840 µm
in the sagittal focusing direction	450 µm
Numerical aperture:	
in the meridional focusing direction	0.0021
in the sagittal focusing direction	0.0011
Diffraction-limited focus size at 7-keV X-ray:	
in the meridional focusing direction	37 nm (FWHM)
in the sagittal focusing direction	67 nm (FWHM)

**Figure 2:**
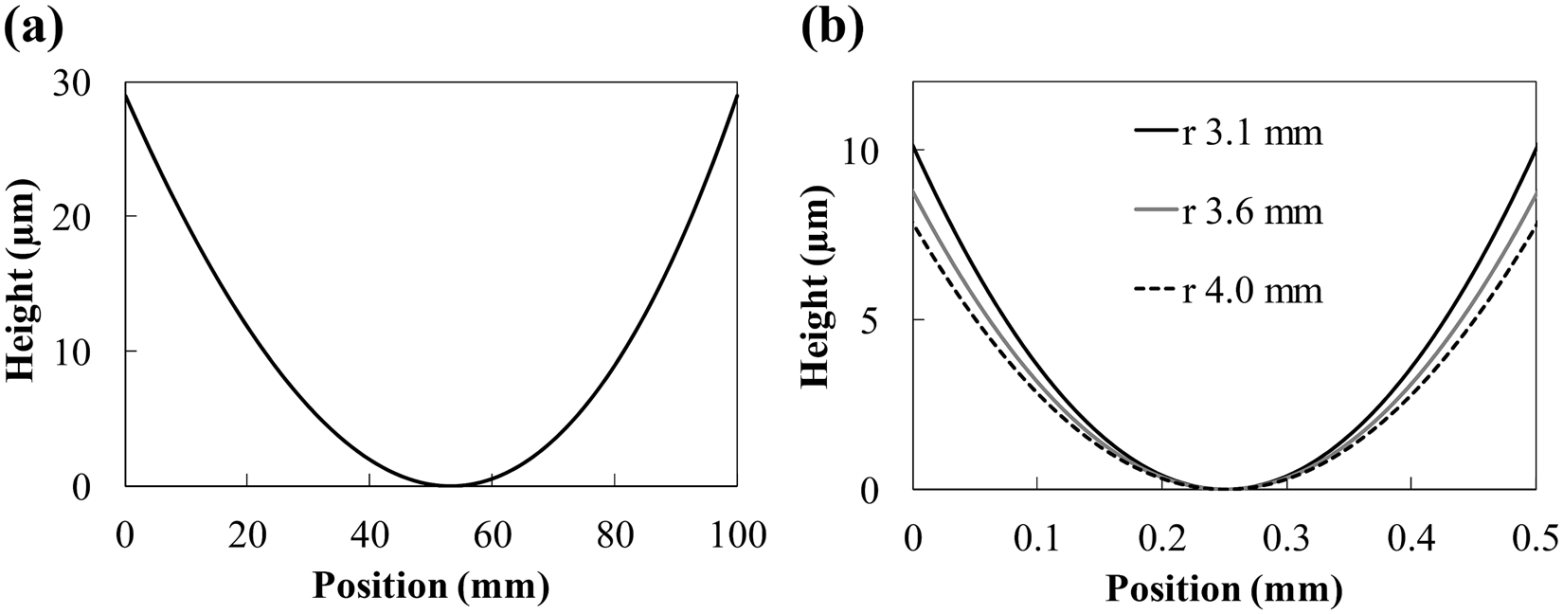
Surface profiles of designed ellipsoidal mirror. (**a**) Surface profile in the meridional focusing direction. (**b**) Surface profiles in the sagittal focusing direction. Three profiles with radii of curvature of 3.1, 3.6, and 4.0 mm correspond to the mirror surface at the downstream edge, center, and upstream edge in the meridional focusing direction.

Figure 2 shows the designed surface profiles of the ellipsoidal mirror in the meridional and sagittal focusing directions. The radius of curvature of the surface profile is 29–62 m and 3.1–4.0 mm in the meridional and sagittal focusing directions, respectively. The relationship between the incident angle *θ* and the radius of curvature in the meridional and sagittal focusing directions, *R*_*m*_ and *R*_*s*_, respectively, is expressed^4,31^ as *R*_*s*_ ≈ *R*_*m*_ *θ* ^2^. Thus, *R*_*s*_ is 4–6 orders of magnitude lesser than *R*_*m*_ in the hard X-ray region under grazing-incidence angle conditions. We manufactured the ellipsoidal focusing mirror by using the process presented in the Methods section (“Manufacturing process of ellipsoidal focusing mirror” subsection) that is applicable to a small radius of curvature^31,32,35^.

**Figure 3:**
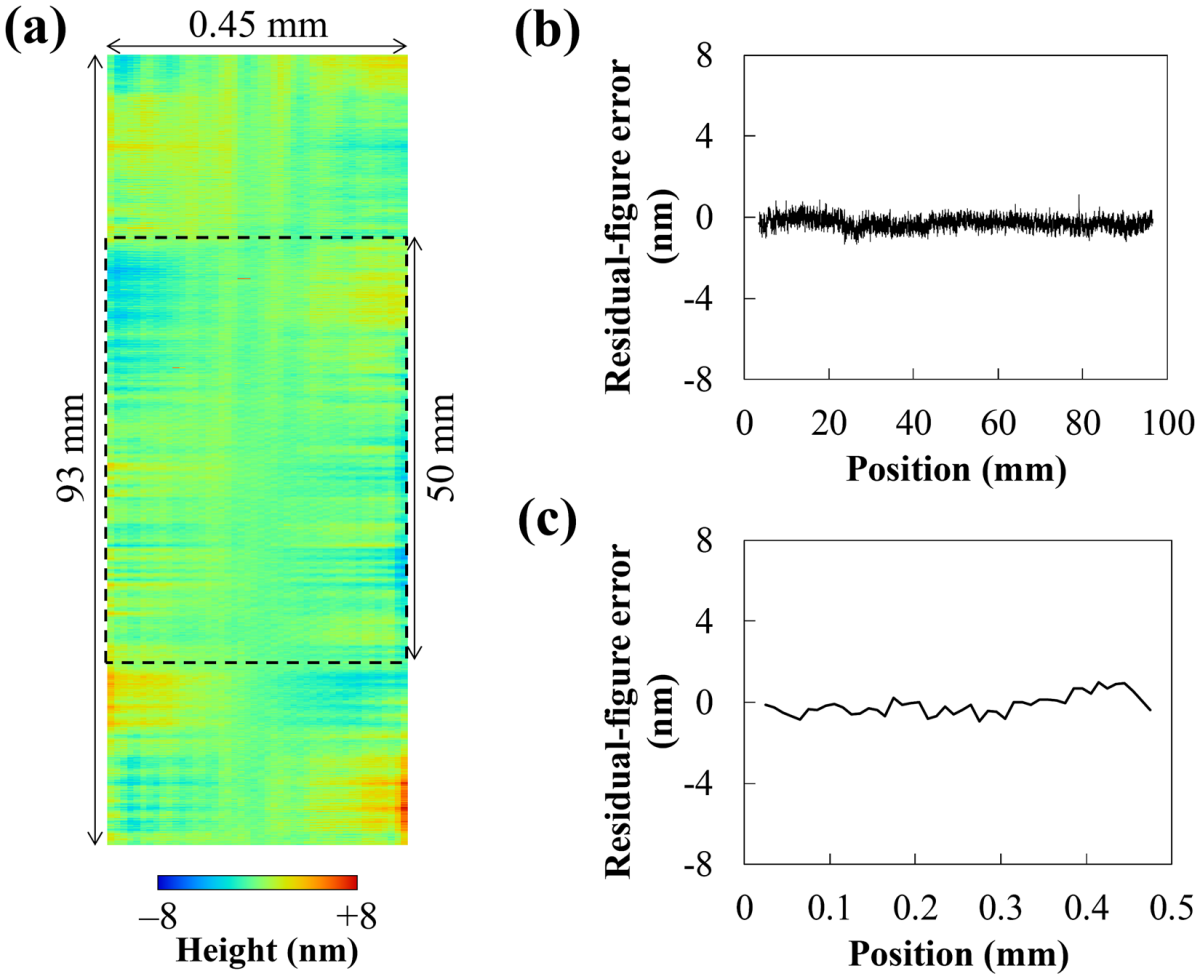
Residual-figure errors of manufactured ellipsoidal focusing mirror. (**a**) Two-dimensional height map of the residual-figure errors. (**b**) and (**c**) Cross-sectional profiles along the center line in the meridional and sagittal focusing directions, respectively. Residual-figure error at the evaluated dimension of 93 mm × 0.45 mm is 1.0 nm (RMS). Residual-figure error at the evaluated dimension of 50 mm × 0.45 mm, indicated by the dashed rectangle in (**a**), is 0.8 nm (RMS).

Figure 3 shows the manufacturing results of the ellipsoidal mirror. This mirror was measured using our developed stitching method^32^. We finished the whole region of the designed mirror surface. Figure 3(a) shows a two-dimensional height map of the residual-figure errors; this is the difference of the measured shape from the designed ellipsoidal shape. Figure 3(b) and (c) show the cross-sectional profiles along the center line in the meridional and sagittal focusing directions shown in Fig. 3(a). The mirror center had a depth of 7 µm and a slope distribution of ±63 mrad along the 0.45-mm length in the sagittal focusing direction. The residual-figure error for evaluated dimensions of 93 mm × 0.45 mm was 1.0 nm (RMS); this is comparable to the measurement reproducibility and required surface accuracy. The central-part area with dimensions of 50 mm × 0.45 mm, indicated by the dashed rectangle in Fig. 3(a), had a residual-figure error of 0.8 nm (RMS).

### Hard X-ray nanofocusing using ellipsoidal mirror

**Figure 4:**
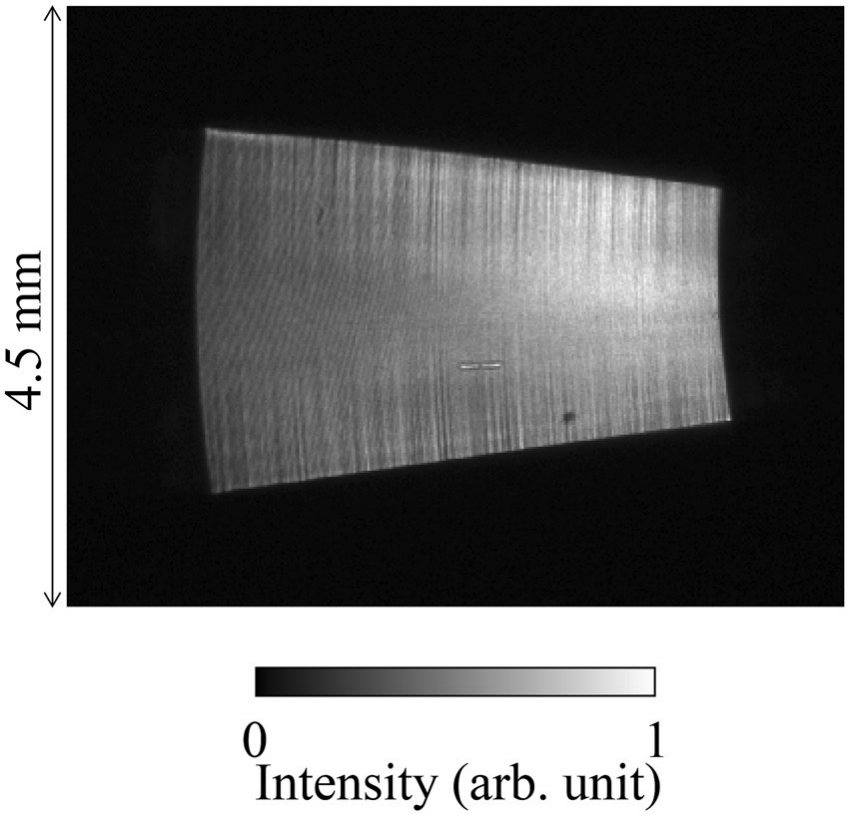
Intensity distribution reflected from ellipsoidal focusing mirror. The image is observed at X-ray energy of 7 keV using a two-dimensional X-ray detector located 0.9 m downstream from the focus. The vertical and horizontal directions in the figure correspond to the sagittal and meridional focusing directions.

We evaluated the focusing properties of the developed ellipsoidal mirror in the synchrotron-radiation facility of SPring-8 (see “Testing at a synchrotron-radiation beamline” subsection in the Methods section). The focus sizes were mainly measured to determine whether the manufacturing process can realize two-dimensional 100-nm focusing. Figure 4 shows a reflected image measured using a two-dimensional detector (C10600-10B-H and AA20MOD, Hamamatsu Photonics K.K.) at X-ray energy of 7 keV. The vertical and horizontal directions in the figure correspond to the sagittal and meridional focusing directions, respectively. Because each point on the ellipsoidal surface had different focal lengths along the meridional focusing direction, the magnification factor of the ellipsoidal surface along the meridional focusing direction had a different value. Thus, the reflected image from the ellipsoidal mirror had a sectoral form, as shown in Fig. 4.

**Figure 5:**
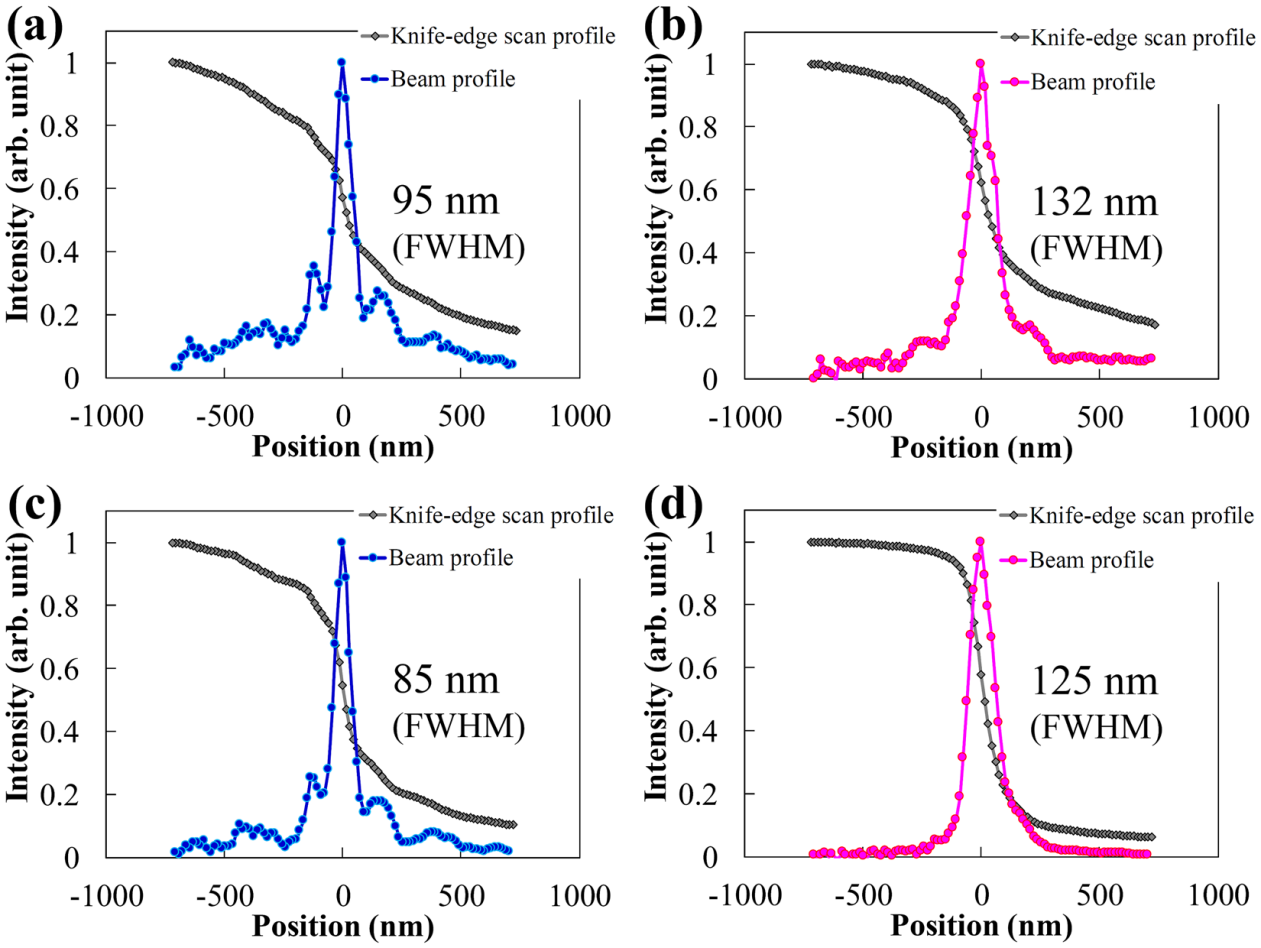
Measured intensity distributions in focal plane. The intensity distributions reflected from two areas of the ellipsoidal mirror are evaluated using the knife-edge scanning method at X-ray energy of 7 keV. One has a total area of 93 mm × 0.45 mm. The other is the 50 mm × 0.45 mm central-part area. The corresponding profiles are shown in (**a**) and (**b**) and in (**c**) and (**d**), respectively. (**a**) and (**c**) Focusing-beam profiles in the meridional focusing direction. (**b**) and (**d**) Focusing-beam profiles in the sagittal focusing direction. The evaluated focus sizes are 95 nm × 132 nm and 85 nm × 125 nm (FWHM), respectively.

The ellipsoidal focusing mirror was aligned precisely using Foucault’s knife-edge test^36^ according to the wave-optical simulation (see “Evaluation of focusing property” subsection in the Methods section). Figure 5 shows the evaluated focusing-beam profiles using an ellipsoidal mirror at X-ray energy of 7 keV. We evaluated two reflective areas on the mirror surface. One had a total area of 93 mm × 0.45 mm, as shown in Fig. 5(a) and (b) and the other was the central-part area of 50 mm × 0.45 mm, as shown in Fig. 5(c) and (d). We achieved focus sizes of 95 nm × 132 nm and 85 nm × 125 nm (FWHM) from these respective reflective areas. Figure 5(a) and (c) show the focusing-beam profiles in the meridional focusing direction, and Fig. 5(b) and (d) show those in the sagittal focusing direction.

**Figure 6:**
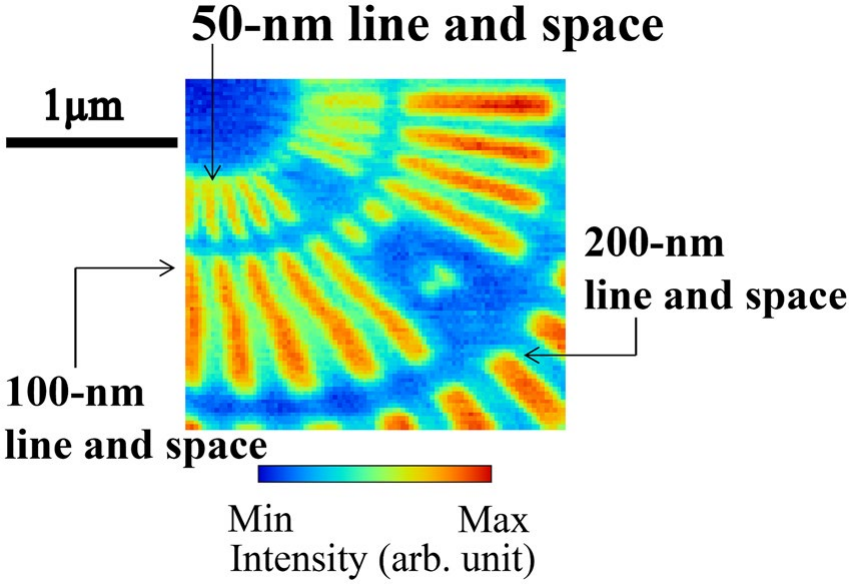
Absorption-contrast X-ray image of resolution-test object observed by scanning X-ray microscopy using generated focusing beam. The vertical and horizontal directions of the image correspond to the sagittal and meridional focusing directions, respectively. The test object shows radial patterns with minimum line and space structures of 50 nm. The image consists of 90 × 83 pixels in total with a pixel width of 25 nm.

For evaluating astigmatism, a two-dimensional-resolution test object was observed by scanning X-ray microscopy by using the generated focusing beam (see “Scanning X-ray microscopy” subsection in the Methods section). Figure 6 shows an obtained absorption-contrast X-ray image of the test object with measurement dimensions of 2.2 µm × 2.1 µm. The image consists of 90 × 83 pixels in total, with pixel width of 25 nm. The vertical and horizontal directions of the image corresponded to the sagittal and meridional focusing directions, respectively. The resolution test pattern of the 100-nm line and space structures were clearly resolved in both the meridional and the sagittal focusing directions. In addition, the 50-nm line and space structures were resolved in both directions as designed.

## Discussion

The measured focus size reflected by the total area was larger than the estimated value, whereas the reflection image from the mirror was relatively clean. The measured minimum focus size reflected by the central-part area was 1.2–1.8 times larger than the diffraction-limited focus size of 70 nm (meridional) × 69 nm (sagittal) (FWHM); this was estimated by the wave-optical simulator using the corresponding mirror aperture. The observed focus sizes from the total area were larger than that from the central-part area. Even though a small measurement error could occur in the profile measurements when using the knife-edge scanning method owing to scattering from the surface of the knife edge, the most likely cause of the increase in focus sizes is the lack of absolute accuracy of the surface metrology rather than the alignment error of the mirror. This result is based on the observed conditions in Foucault’s knife-edge test in the measurement. The intensity around the main peak in the meridional focusing direction was higher than that in the sagittal focusing direction. This would be most likely due to a difference in the surface figure accuracy of the ellipsoidal focusing mirror between the focusing directions. Measurement errors (systematic errors) tend to occur in the stitching direction that corresponds to the meridional focusing direction of the ellipsoidal mirror. To determine the surface accuracy of the mirror more precisely, we need more accurate X-ray tests using wavefront assessment methods such as phytography^37,38^ and grating interferometry^13,39^. The focusing properties of ellipsoidal mirrors will be improved by enhanced surface metrology and adaptive optics^38^ in future works. Nevertheless, the observations indicated that two-dimensional 100-nm focusing was obviously achieved in the same focal plane without astigmatism over the measurement time under exactly aligned conditions. We believe that the realization of ellipsoidal focusing mirror by resolving two-dimensional 50-nm structures will open up new opportunities for extensive applications.

Our achievements can contribute to wide-ranging scientific applications. Their significance is summarized in terms of two main improvements: (1) availability of highly efficient ellipsoidal focusing mirror and (2) future possibilities for developing novel optics based on technological advancements in the manufacturing processes of precision two-dimensional aspheric mirrors with a highly sloped surface.

The developed manufacturing technologies can be used to create ellipsoidal mirrors that offer advantages for use from conventional to advanced optics in X-ray applications. In particular, the high-flux and high-density beam from ellipsoidal focusing mirrors can be applied to photon-hungry experiments^40–43^ that do not always need nanofocusing performance but do require the highest possible flux on a small sample in synchrotron-radiation and XFEL facilities.

**Figure 7:**
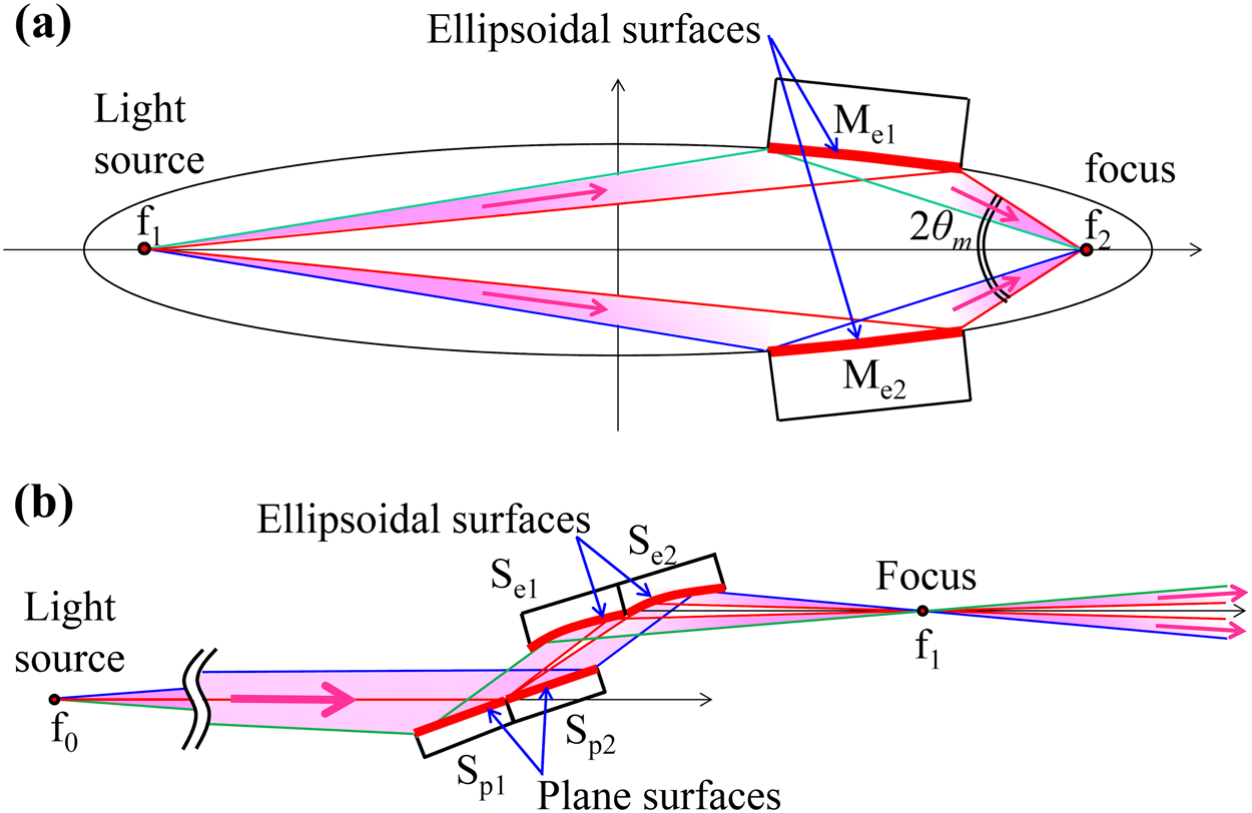
Layout of focusing optics using ellipsoidal mirrors. (**a**) Focusing optical system with a large N.A. X-rays from the light source (f_1_) are reflected by two ellipsoidal focusing mirrors (M_e1_ and M_e2_) that face each other. Then, the reflected X-rays are two-dimensionally focused at one focal point (f_2_). This optics can be designed to have a small diffraction-limited focus size compared with the focusing optics that uses a single ellipsoidal mirror, as a result of the large N.A. For example, N.A. is 0.0207, which is calculated from a convergence angle (2*θ*_*m*_) of 41.5 mrad, when the ellipsoidal focusing mirrors designed in the present work are used. The diffraction-limited focus size (*δ*) can be roughly estimated in the present case using the well-known formula^59^ *δ* = 0.61 × *λ*/N.A., where *λ* is the wavelength of light. Therefore, *δ* is calculated as 5.2 and 1.8 nm (FWHM) when *λ* is 0.177 nm (7 keV) in the case of the total reflection mirrors and 0.062 nm (20 keV) in the case of multilayer mirrors, respectively. (**b**) Upstream optics for the (**a**) optical system to produce two divergent beams. The X-rays from the primary light source (f_0_) are reflected and divided into two beams by the two plane surfaces (S_p1_ and S_p2_) with different incident angles. Then, the two beams are two-dimensionally focused at point f_1_ by the two ellipsoidal surfaces (S_e1_ and S_e2_) and become two divergent beams. The two-step focusing optics with the (**b**) upstream and (**a**) downstream optics can be designed using common focus f_1_ as a secondary light source for the (**a**) optical system. However, the intensities in the core region of the incident beam are lost when one large incident beam illuminates the two ellipsoidal mirrors (M_e1_ and M_e2_) in (**a**) the optical system. This event can be avoided using (**b**) the optical system with two divergent beams. In addition, the two-step focusing optics can have a large demagnification ratio, which is essential for nanofocusing optics.

Our technological advancements in the manufacturing process can improve the optical design flexibility, such as for the Wolter mirror optics that uses ellipsoidal and hyperboloidal mirrors^44,45^, our proposed new focusing optics (see Fig. 7, for example), and others^46,47^. These optical arrangements with ideal properties were impossible thus far owing to the difficulties faced in manufacturing two-dimensional aspherical mirrors. Figure 7 shows high-efficiency nanofocusing optics using ellipsoidal mirrors that can be applied in synchrotron radiation and XFEL facilities. This optics enables a highly intense nanofocusing beam by taking advantage of two-step and two-beam optics using two-dimensional focusing mirrors (see legend for details). The precision manufacturing technologies for two-dimensional aspherical mirrors can produce optics not only for current synchrotron-radiation and XFEL facilities but also for other scientific fields for future low-emittance synchrotron-radiation facilities^48^, XFEL oscillators^49^, and astrometric telescopes^50^. In addition, ellipsoidal focusing mirrors with a multilayer coating can potentially realize a focus size of nearly 1 nm with a long working distance and a high flux owing to the wide energy bandwidth and large spatial aperture^51,52^ (see Fig. 7), in contrast to other optics that realized approximately 10-nm focusing property^53–57^.

## Methods

### Properties of ellipsoidal focusing mirror

The mirror has a long working distance of 150 mm from the downstream edge of the mirror to the focus. This long distance enables device settings such as detecting signals from samples and changing sample environments, and it is beneficial for microscopic applications. The focus sizes of 38 nm (meridional) × 81 nm (sagittal) (FWHM) for the total area and 73 nm (meridional) × 75 nm (sagittal) (FWHM) for the central-part area were estimated using a wave-optical simulator in which the residual-figure errors measured using the stitching method were considered. The focus size in the sagittal direction seemed to be more sensitive to the measured residual-figure errors. The reason for the difference is that the accuracy of stitching angles in the sagittal direction is lower than that in the meridional direction owing to a measurement setup. The measurement repeatability limits the sagittal focus size.

Many favorable characteristics of ellipsoidal mirrors that are important in microscopic applications of optics arise from the change from two-mirror to one-mirror optics. These characteristics include the following: (1) improvement in focusing efficiency with a long working distance that results from an optical design with high demagnification ratio and avoidance of reflectance loss from the mirror, (2) simplification of the mirror alignment mechanism in terms of the size and number of controlled axes, (3) stability improvement of the focus size and position, and (4) reduction in the deflection angles relative to the incident X-rays. This advantage facilitates the handling of the reflected focusing beam.

### Manufacturing process of ellipsoidal focusing mirror

Initially, a mirror substrate was efficiently and roughly formed into a desired ellipsoidal shape by using multiaxis precision grinding with a height accuracy of ~1 µm (PV). Then, the surface roughness was improved to an X-ray mirror quality of 0.2 nm (RMS). Next, the surface-figure errors were eliminated using a computer-controlled figure-correction method to meet the required figure accuracy. Finally, the mirror surface was uniformly coated with platinum by a DC magnetron sputtering method. The surface roughness of the finished surface after platinum coating was 0.3 nm (RMS) under the evaluated dimension of 0.1 mm × 0.1 mm.

For the manufacturing process of ellipsoidal mirrors, we developed unique surface-finishing technologies^35^ and surface metrology^32^. Both surface-finishing technologies for improvement of the surface roughness and figure correction were based on an ultraprecision machining method, namely, elastic emission machining^22,23^. For reducing the residual-figure errors in the mirror surface, a nozzle-jet-type machine^35^ with an aperture size of 50 µm was used. This machine can produce a removal spot with a size of 80 µm (FWHM). Intended materials on a workpiece surface can be removed by supplying locally fine-particle slurry via the small nozzle. The figure correction process is conducted to remove local residual-figure errors by changing the scanning speed of the nozzle head relative to the workpiece on the basis of the surface metrology.

Surface metrology limits the accuracy of computer-controlled figure correction, that is, it limits the final accuracy. Surface metrology was based on precision stitching interferometry of the relative angle determinable stitching interferometry^25,26^ using a specialized correction method for a highly sloped surface^32^. The surface metrology used in the manufacturing limits the sagittal numerical aperture. The measurable maximum slope in subaperture measurements by a microscopic interferometer was ~80 mrad under existing surface metrology conditions (a 20× objective, a 0.5× zoom lens, and a camera with 640 × 480 resolution array)^32^. The manufactured mirror had a maximum slope of 80 mrad.

### Testing at a synchrotron-radiation beamline

The assessment was conducted at Experimental Hutch 3, BL29XUL^58^, of an undulator source beamline. Monochromatic X-rays from a Si (111) double-crystal monochromator were supplied to this beamline. A 5 µm × 5 µm rectangular aperture with crossed slits was used as a secondary light source, and it was set 50 m upstream from the mirror to produce a sufficiently small geometric focus size. Thus, the source slit can be considered a point source in this optics. This is useful for evaluating the focus size by illuminating the mirror under almost ideal conditions. However, it is not suitable for obtaining a high focusing flux. To obtain a high flux focus using the ellipsoidal mirror with 100-nm focus size, we need additional optimized optics such as another focusing optics (two-step focus) to strongly illuminate the secondary light source.

### Evaluation of focusing property

After precision alignment of the incident angle, in-plane rotation angle, and focal length, we measured the focusing-intensity distribution using a knife-edge scanning method. We used a 200-µm-diameter gold wire as the knife edge and the two-axis positioning stage of a 1-nm feedback stage system (FS-1040SPXY(MD), Sigma Tech. Co., Ltd.). This motorized stage provides positioning resolution of 1 nm via closed-loop feedback control with reference to a linear scale.

### Scanning X-ray microscopy

The observed test object was made of 500-nm-thick tantalum and had radial patterns with minimum line and space structures of 50 nm (XRESO-50HC, NTT-AT Corp.). The test structure was scanned using the 1-nm feedback stage system with exposure time of 0.1 s/pixel. The measurement took about 0.8 s/pixel to detect the X-ray intensity and to move the scanning stage. The acquisition time of the image (Fig. 6) was 1.8 h. A long acquisition time of 1 h was selected to examine the stability of the two-dimensional focus. The X-ray intensity passing through the test structure was measured using a silicon PIN photodiode detector. At the same time, the intensity fluctuation of the incident X-rays was measured using an ionization chamber for normalization.

### Data availability

The datasets generated and/or analyzed during the current study are available from the corresponding author upon reasonable request.
